# Spatiotemporal gait characteristics during single- and dual-task walking are associated with the burden of cerebral small vessel disease

**DOI:** 10.3389/fneur.2023.1285947

**Published:** 2023-11-13

**Authors:** Cuiqiao Xia, Hongyang Xie, Tianjiao Li, Yu Ding, Hóngyi Zhào, Yonghua Huang

**Affiliations:** ^1^Department of Neurology, Chinese PLA General Hospital, Beijing, China; ^2^Outpatient Department of Haidian No. 58 Retired Cadre Rest Centre of the PLA, Beijing, China; ^3^Department of Neurology, Number 984 Hospital of the PLA, Beijing, China

**Keywords:** cerebral small vessel disease, gait, dual-task walking, white matter hyperintensity, neuroimaging biomarkers

## Abstract

**Introduction:**

Gait impairment is a common symptom among individuals with cerebral small vessel disease (CSVD). However, performance differences between single-task walking (STW) and dual-task walking (DTW) among individuals with CSVD remain unclear. Therefore, we aimed to examine differences in gait characteristics during STW and DTW as well as the association between gait performance and neuroimaging markers.

**Methods:**

We enrolled 126 older individuals with CSVD. The speed, cadence, stride length, stride time, and their dual-task cost (DTC) or variability were measured under the STW, motor-cognitive DTW (cognitive DTW), and motor-motor DTW (motor DTW) conditions. We examined neuroimaging features such as white matter hyperintensities (WMHs), lacunes, microbleeds, and total burden. Further, we analysed the association of neuroimaging markers with gait performance, including gait variability and DTC.

**Results:**

Almost all spatiotemporal characteristics, as well as their DTCs or variabilities, showed significant among-group differences according to disease severity in the cognitive DTW condition; however, relatively lesser differences were observed in the STW and motor DTW conditions. The total CSVD burden score was moderately correlated with all the spatial parameters, as well as their DTCs or variabilities, in the cognitive DTW condition. Moreover, WMHs showed a correlation with speed, stride time, and cadence, as well as their DTCs, in the cognitive DTW condition. Furthermore, lacunes showed a moderate correlation with speed, stride length, and the DTC of speed, whilst microbleeds were only related to the DTC of stride length in the cognitive DTW condition. Neuroimaging biomarkers were not correlated with spatiotemporal parameters in STW and motor DTW conditions after Bonferroni correction. Moreover, the correlation coefficient between the total CSVD burden score and gait parameters was greater than those of other biomarkers.

**Discussion:**

Parameters in the cognitive DTW condition are more appropriate than those in the motor DTW condition for the evaluation of gait abnormalities in patients with CSVD. Moreover, the total CSVD burden score might have better predictive utility than any single neuroimaging marker. Patients with CSVD, especially those with moderate-to-severe disease, should concentrate more on their gait patterns and reduce the load of secondary cognitive tasks whilst walking in daily life.

## 1. Introduction

Dual-task walking (DTW) is defined as walking whilst performing a secondary task, including a cognitive task (motor-cognitive DTW) or motor task (motor-motor DTW) (1). In daily life, people perform various tasks, including speaking or picking up a coffee mug, whilst walking. Therefore, DTW offers an appropriate method for assessing gait characteristics in daily life (2). Healthy individuals can simultaneously complete several tasks; however, this can be difficult for patients with neurological disorders. Accordingly, patients with vascular or neurodegenerative diseases are more likely to present deteriorated walking patterns during DTW than during single-task walking (STW) (3, 4). However, the gait characteristics of DTW among individuals with cerebral small vessel disease (CSVD) remain unclear (5, 6). Walking (which involves the planning and execution of movements, postural control, and motor coordination) and the secondary task would simultaneously compete for similar neural resources. Patients with diseases might have a reduced total of neural resources available, and this loss may be compensated during STW; however, this compensatory mechanism is limited by the secondary task during DTW. Motor-cognitive DTW (cognitive DTW) and motor-motor DTW (motor DTW) can be achieved by asking participants to perform arithmetic calculations (7–9) and carry a tray of empty glasses (8, 10), respectively, whilst walking.

In CSVD, structural abnormalities or dysfunction of cerebral microvessels often results in clinical, imaging, or pathological changes (11). Generally, CSVD affects the integrity of white matter connectivity (12) and is detected via brain computed tomography or magnetic resonance imaging (MRI). Imaging-based biomarkers for CSVD include recent small subcortical infarcts, white matter hyperintensities (WMHs), lacunar infarctions, enlarged perivascular spaces, cerebral microbleeds, and atrophy (13). The clinical characters of CSVD are gait abnormalities and cognitive deficits, which increase the risk of falls and mortality (14). Further, CSVD and gait disorders have several potential common mechanisms. For example, WMHs may influence gait posture through alterations in motion pathways and cortical thickness, changes in cognition and motion pathways induced by lacunar infarctions may exacerbate gait disorders, and damage to motion pathways induced by cerebral microbleeds may affect gait (15). Furthermore, the primary cognitive impairment observed in CSVD is related to executive function (14). This is also supported by the documented correlation between gait speed and executive function performance in the elderly (16). Consequently, the lack of executive function leads to gait deterioration in patients with CSVD. Moreover, brain regions involved in gait control and cognitive tasks appear to be interlinked (17). Specifically, the sensorimotor network, frontoparietal network, and auxiliary motor areas might influence gait control and cognitive tasks simultaneously (18). Gait might be interfered when the concurrent tasks compete for these shared neural networks which is limited in CSVD patients (19). Furthermore, Coleman's work verified a 1:1 integer ratio between heart and gait rates in normal individuals (20). Coupling between cardiac and locomotor rhythms is present at faster speeds but not at slow or comfortable speeds in healthy individuals. This suggests that the two rhythms must couple when there is a need to compensate for cognitive difficulties, such as during a subtraction task (21). However, this compensatory mechanism may be limited in certain diseases. Additionally, heart rate variability is related to executive function and prefrontal neural function, which play a critical role in the performance of DTW (19, 22). Therefore, the change in cardiac rhythm might be another reason for the deterioration in DTW performance. As a systemic disease, CSVD can impair the function of multiple organs, particularly the heart. The study indicates that decreased heart rate variability may be a potential risk factor for CSVD (23). Additionally, in most cases, individuals with CSVD do not often present with clinical abnormalities; instead, they show atypical neurological symptoms that are difficult to detect using traditional methods. DTW can exacerbate gait disorders and provide vital information regarding changes in walking patterns. Therefore, patients with CSVD may be particularly affected by DTW. Moreover, digital gait analysis can provide accurate parameters for assessing gait disorders (24).

Motor and cognitive DTW conditions have been widely used in studies on neurological diseases, including CSVD (5, 6, 9). However, there remains no consensus on which secondary task should be paired with walking to detect changes in gait. Accordingly, we aimed to investigate differences in walking patterns between motor and cognitive DTW. Further, we assessed the association of neuroimaging biomarkers with gait characteristics during STW and DTW. According to previously observation in our hospital, the hypotheses were as follows: (1) the total CSVD burden score may have superior predictive validity and (2) cognitive DTW may play an important role in detecting gait deterioration.

## 2. Materials and methods

### 2.1. Participants

This study was conducted in accordance with the Declaration of Helsinki and approved by the Academic Ethics Committee of the Biological Sciences Division of the Seventh Medical Centre of the People's Liberation Army General Hospital (Beijing, China). All participants provided informed consent.

This cross-sectional study included 126 patients with CSVD from the Department of Neurology at the Seventh Medical Centre of People's Liberation Army General Hospital (Beijing, China) from September 1, 2020, to September 1, 2021. All patients were screened and interviewed by research personnel. Most participants were willing to undergo MRI owing to complaints such as mild dizziness, mild headache, mood disturbances, cognitive disorders, and motor dysfunction. The inclusion criteria were as follows: (1) ability to independently walk 31 steps; (2) sufficient ability to comprehend and adhere to instructions; and (3) having at least one of the following imaging manifestations of CSVD: WMH, lacunar infarction, or microbleeds. The exclusion criteria were as follows: (1) inability to independently walk 31 steps with or without a tray in their hands or (2) inability to comprehend and adhere to instructions. Further, we excluded individuals with any of the following characteristics: acute cerebral ischaemic or bleeding episodes; leukoencephalopathy with immune, demyelinating, or genetic causes; major psychiatric diseases; gait and balance disorders caused by non-vascular factors such as degenerative or musculoskeletal diseases; dementia based on the Mini-Mental State Examination (MMSE) score (25), and the optimal cut-off points for dementia were 17 for illiterate, 20 for individuals with 1–6 years of education, and 24 for individuals with seven or more years of education (26); communication barriers, including language, vision, or hearing impairment that could impede assessment efforts; and MRI contraindications. The age, sex, years of education, and MMSE scores were recorded. Additionally, the number of comorbid conditions (hypertension, angina, myocardial infarction, hyperlipidaemia, diabetes, migraine, and arthritis), as well as medical and treatment histories, were recorded during in-person interviews.

### 2.2. MRI measurements and total CSVD score

Neuroimaging parameters were obtained using a 3.0 T MRI scanner (SIEMENS AG, Munich, Germany). The imaging protocols were as follows: T1-weighted imaging, T2-weighted imaging, fluid-attenuated inversion recovery, and susceptibility-weighted imaging. The findings were interpreted by two experienced clinical neurologists who were blinded to information regarding the participants.

Lacunar infarcts are often defined as round or ovoid, subcortical, fluid-filled cavities (signal similar to that of cerebrospinal fluid) with a diameter of 3–15 mm and are consistent with a previous acute small subcortical infarct or haemorrhage (13). Contrastingly, microbleeds are defined as small (generally 2–5 mm in diameter but sometimes up to 10 mm) signal-void areas with associated blooming observed on T2-weighted or susceptibility-weighted imaging (13). WMH were assessed using the Fazekas scale (27). The highest grade observed in either the periventricular or subcortical area (grades 1–3) was documented (9, 27). The Fazekas scale is a simple tool that is widely used in studies on CSVD and Alzheimer's disease; moreover, the number of lacunar infarcts and microbleeds was calculated. These parameters were used to determine the total CSVD burden score, which may better reflect the overall CSVD-induced damage than a single neuroimaging biomarker (28). Specifically, the total CSVD burden score was calculated by counting the number of MRI features, including lacunar infarcts, cerebral microbleeds, and WMHs (score range: 0–3), as previously described (28).

Here, one point was assigned if the participant had three or more lacunar infarctions. Another point was assigned if the participant had any number of cerebral microbleeds. Finally, another point was added if the participant showed confluent or deep WMHs (Fazekas score: 2 or 3). As only a few participants attained the highest total CSVD burden scores, the participants were divided into the following three subgroups: high burden (score: 2 or 3), medium burden (score: 1), and low burden (score: 0). Additionally, when lacunes or microbleeds were calculated separately, the existence of lacunes or microbleeds was defined as the presence of two or more lacunes or any microbleeds.

### 2.3. STW and DTW methods

Gait parameters were obtained from patients under different conditions: (1) only walking (STW), (2) walking whilst performing three serial subtractions from a randomly selected starting number (90, 95, 100, or 105; cognitive DTW). For example, 95-3,−3, and−3; and (3) walking whilst holding a tray with four empty glasses placed on the corners (motor DTW).

A meta-analysis indicated that cognitive tasks that involve internal interfering factors (mental tracking) seem to disturb gait performance more than those involving external interfering factors (reaction time) (29). Another literature review of Parkinson′s disease showed that secondary tasks which mainly involved executive functions, including arithmetic, language, or motor functions, considerably affected gait parameters; however, tasks that involved memory and visual-spatial function only had a small effect on gait characteristics (4). Moreover, although studies used different secondary tasks for DTW, there was strong evidence suggesting that velocity was affected in DTW across all the secondary tasks (4). Given the simplicity and accessibility of arithmetic tasks, we applied it to the cognitive DTW tasks.

In both STW and DTW conditions, the participants comfortably walked 31 strides in a passageway. According to the regulations, participants are required to commence walking 6 steps before the starting line and conclude six steps after the 25th stride. To prevent the impact of acceleration and deceleration on gait, we only analysed 25 strides in the middle part of the test. In the DTW condition, the participants were required to perform both the walking and secondary tasks to the best of their ability without prioritising either activity. The starting and end points of each trial were marked and recorded.

### 2.4. Measurement methods and gait parameters

Gait parameters were recorded using a MiniSun Intelligent Device for Energy Expenditure and Activity System (MiniSun, Fresno, CA, USA), which is a wearable gait analyser that provides temporal (time) and spatial (distance) information via a movable sensor connected to a computer. It comprises a recorder and five acceleration sensors. The position of the acceleration sensors is fixed on the posterior edge of the fourth metatarsal head on both soles, the anterior and middle positions of the thighs on both sides, and 4 cm below the midpoint of the sternoclavicular joint connexion on both sides. This device has been shown to obtain extremely accurate spatiotemporal parameters at different conditions (walking, running, or climbing up and down stairs) (24, 30–32). Additionally, this device has been used to assess gait disorders in patients with CSVD (9, 33).

We measured the average values of velocity, cadence, stride length, and stride time. Stride time (s) was defined as the time taken to complete a walking cycle, i.e., the time elapsed from the heel of one lower limb leaving the ground to the same heel returning to the ground. Stride length (m) was defined as the distance between the heel of one lower limb leaving the ground to the same heel landing again. Velocity (m/s) was calculated as the velocity of two successive strides. Cadence (steps/min) was calculated as the number of steps/stairs per minute (8).

Gait variability (%) was determined as the coefficient of variation (CV) calculated using the four aforementioned indicators (2). As the ratio of both legs was related, we calculated the CV for one side as follows:


$$\begin{array}{l} stride\;CV=\frac{stdev\left(stride\right)}{mean\left(stride\right)}\times 100\% \end{array}$$

A high CV value indicated large variability. Further, we calculated the dual-task cost (DTC) as follows (34):


$$\begin{array}{l} DTC=-\frac{dual\;task\;value-single\;task\;value}{single\;task\;value\;} \end{array}$$

A positive DTC value indicated lower dual-task ability, whilst a negative value indicated higher dual-task ability in velocity, cadence, and stride length. On the contrary, a negative DTC value indicated lower dual-task ability in stride time.

### 2.5. Neuropsychological assessment

All individuals completed a set of neuropsychological assessments, including the MMSE, verbal fluency test, clock drawing test, and the trail-making test part B (TMT-B). The verbal fluency test was used to assess language skills. Here, the number of animals which could be named by participants within 1 min was counted. The higher the number of names, the less severe the damage to word fluency. Visual search speed and cognitive flexibility were assessed using the TMT-B. Here, the participant connected a trail through symbols (numbers 1, 2, 3, … 9 and corresponding Chinese characters 壹, 贰, 叁, …玖) in the correct order. The time from the instruction to start until trial completion was recorded. The longer the time, the more severe the damage to performing function. Visual-spatial skills were assessed using the transformed score of the clock drawing test. Here, the participants were asked to draw the face of a clock with all the numbers and set the time to 10 min past 11 o'clock. The drawing score system was assessed using 13-point criteria (35, 36). General cognitive function was assessed using the MMSE. A low score indicated limited cognitive function.

### 2.6. Statistical analysis

Normality was assessed using the Shapiro–Wilk test. Variables with normal and skewed distributions are presented as the mean ± standard deviation (SD) and median with interquartile range (IQR), respectively. Categorical variables are represented as frequencies. Normally distributed variables were assessed using a one-way analysis of variance test. Parameters with skewed distributions were analysed using the nonparametric Kruskal–Wallis test. Categorical variables were analysed using the chi-square test. Spatiotemporal parameters and their DTCs or variability were analysed using generalised estimating equations, with three groups (low, medium, and high burden) and two or three conditions in STW and DTW. In the case of interaction between group and condition, we performed the *post-hoc* analysis with Bonferroni correction to detect discrepancies. Spearman's rank correlation was performed to determine the association of neuroimaging markers (ordered categorical variables) with spatiotemporal parameters in different conditions. All statistical analyses were performed using SPSS version 26.0 (IBM, Armonk, NY, USA), all the figures were created with GraphPad Prism 9. Statistical significance was set at *P* < 0.05.

## 3. Results

### 3.1. Demographic characteristics, neuropsychological assessment and neuroimage characteristics

Table 1 presents the basic characteristics of the included participants. We observed no significant among-group differences in age, sex, years of education, or comorbidity (low-, medium-, and high-burden groups; *P* > 0.05). Further, Table 1 summarises the results of traditional cognitive assessment. The total CSVD burden score was negatively correlated with the TMT-B scores. However, there were no among-group differences in the MMSE, clock drawing test, and verbal fluency test scores.

**Table 1:** Demographic and clinical characteristics of patients.

**Variable**	**Low burden (*n* = 30)**	**Medium burden (*n* = 34)**	**High burden (*n* = 62)**	***P*-value**
**Sex (** ***n*** **, %)**				
Male	21 (70.0%)	18 (52.9%)	38 (61.3%)	0.374^‡^
Education (years)	12 (9, 12.8)	12 (9, 12)	12 (9, 12)	0.850^§^
Age (years)	62.6 ± 7.8	66.8 ± 8.3	66.0 ± 9.1	0.123^†^
MMSE (points)	28 (27, 30)	29 (27.8, 30)	29 (27, 30)	0.859^§^
Hypertension (*n*, %)	24 (80.0%)	21 (61.8%)	44 (71.0%)	0.278^‡^
Stenocardia (*n*, %)	3 (10.0%)	6 (17.6%)	3 (4.8%)	0.123^‡^
Myocardial infarct (*n*, %)	2 (6.7%)	4 (11.8%)	5 (8.1%)	0.745^‡^
Hyperlipidaemia (*n*, %)	22 (73.3%)	24 (70.6%)	52 (83.9%)	0.260^‡^
Diabetes (*n*, %)	12 (40.0%)	8 (23.5%)	18 (29.0%)	0.345^‡^
CDT (points)	12 (11, 13)	12 (11, 13)	12 (10, 13)	0.133^§^
TMTB (s)	72.5 (57.8, 84.5)	77.5 (59.8, 84.3)	87.0 (65.0, 97.3)	0.007^§^^**^
VFT (numbers)	17.6 ± 3.6	17.8 ± 3.5	16.3 ± 4.3	0.145^†^

CDT, clock drawing test; MMSE, mini-mental state examination; TMT-B, trail-making test-part B; VFT, verbal fluency test.

† F value.

‡ χ^2^ value.

§ H value.

** P < 0.01.

Additionally, the average number of participants was 2.37 for lacunar infarctions and 1.55 for cerebral microbleeds. Approximately 40 participants exhibited three or more lacunar infarctions, while 60 participants displayed one or more cerebral microbleeds. Furthermore, there were 38 participants with a Fazekas grade of 2 and 20 participants with a grade of 3. Therefore, in accordance with regulations, the participants were divided into groups based on burden level: 30 in the low burden group, 34 in the medium burden group, and 62 in the high burden group.

### 3.2. Changes in spatiotemporal measures and DTC

Tables 2, 3 present the main effects and interactions in spatiotemporal measures and their DTCs or variabilities. The outcomes presented in Table 2 show that there were significant interactions between the group and condition for stride time, stride length, and their variabilities. Moreover, speed and cadence had a significant interaction; however, only significant main effects were observed for the variabilities in speed and cadence. Therefore, we included all variables in the *post-hoc* analysis, except for the variabilities in speed and cadence. In the STW condition, only speed and stride length (*P* = 0.034 and *P* = 0.014, respectively) differed between the low-burden and high-burden groups. Similarly, in the motor DTW condition, there were differences in stride length (*P* = 0.021) between the low-burden and high-burden groups. Notably, almost all parameters showed among-group differences in the cognitive DTW condition (0.026 < *P* < 9.078 × 10^−10^). In the high-burden group, deterioration of spatial parameters during the motor DTW condition was seen compared with those in the STW condition (speed, *P* = 0.005; stride length, *P* = 0.014). Contrastingly, almost all spatiotemporal parameters of cognitive DTW significantly deteriorated compared with those in the STW condition in the low-burden (speed, *P* = 0.024; cadence, *P* = 0.001; stride length, *P* = 0.044; stride time, *P* = 0.086), medium-burden (speed, *P* < 0.001; cadence, *P* < 0.001; stride length, *P* = 0.155; stride time, *P* < 0.001), and high-burden (speed, *P* < 0.001; cadence, *P* < 0.001; stride length, *P* < 0.001; stride time, *P* < 0.001) groups. In addition, we observed significant differences in the CV of stride time (*P* = 0.002) and length (*P* = 0.002) between the STW and cognitive DTW conditions only in the high-burden group.

**Table 2:** Differences in gait parameters in generalised estimation equation among patients with cerebral small vessel disease.

		**Velocity**	**Stride length**	**Cadence**	**Stride time**	**Velocity CV**	**Stride length CV**	**Cadence CV**	**Stride time CV**
**Main effect**									
Group (disease severities)	Wald χ^2^	14.409	13.261	4.770	8.512	12.712	9.725	15.564	11.879
	*P*	0.001^**^	0.001^**^	0.092	0.014^*^	0.002^**^	0.008^**^	< 0.001^**^	0.003^**^
Condition (STW vs. DTW)	Wald χ^2^	88.661	33.029	86.663	85.921	0.312	6.767	10.615	6.507
	*P*	< 0.001^**^	< 0.001^**^	< 0.001^**^	< 0.001^**^	0.855	0.034^*^	0.005^**^	0.039^*^
**Interaction**									
Group × condition	Wald χ^2^	42.947	18.395	23.169	40.159	4.906	12.292	2.410	13.982
	*P*	< 0.001^**^	0.001^**^	< 0.001^**^	< 0.001^**^	0.297	0.015^*^	0.661	0.007^**^

CV, coefficient of variation; DTW, dual-task walking; STW, single-task walking.

* P < 0.05.

** P < 0.01.

The results were corrected using the Bonferroni method.

Group: comparison among degrees of disease severity. Condition: comparison between STW and DTW.

**Table 3:** Differences in gait DTC in the generalised estimation equation among patients with cerebral small vessel disease.

		**Velocity DTC**	**Stride length DTC**	**Cadence DTC**	**Stride time DTC**
**Main effect**					
Group (disease severities)	Wald χ^2^	18.494	16.801	11.838	19.173
	*P*	< 0.001^**^	< 0.001^**^	0.003^**^	< 0.001^**^
Condition (STW vs. DTW)	Wald χ^2^	32.355	17.072	47.719	41.903
	*P*	< 0.001^**^	< 0.001^**^	< 0.001^**^	< 0.001^**^
**Interaction**					
Group × condition	Wald χ^2^	25.423	30.233	19.086	23.378
	*P*	< 0.001^**^	< 0.001^**^	< 0.001^**^	< 0.001^**^

DTC, dual task cost; DTW, dual-task walking; STW, single-task walking.

** P < 0.01.

The results were corrected using the Bonferroni method.

Group: Comparison among degrees of disease severity. Condition: comparison between STW and DTW.

As summarised in Table 3, the DTCs in the motor and cognitive DTW conditions showed significant main effects and interactions for all parameters. *Post-hoc* analyses revealed similar findings as those mentioned above. We observed significant among-group differences in the DTCs for all parameters in the cognitive DTW condition (0.046 < *P* < 2.883 × 10^−12^) but not in the motor DTW condition (*P* > 0.050). Furthermore, compared with the motor DTW condition, the cognitive DTW condition showed significantly higher DTCs in the medium-burden (speed, *P* < 0.001; cadence, *P* < 0.001; stride length, *P* = 0.040; stride time, *P* < 0.001) and high-burden (speed, *P* < 0.001; cadence, *P* < 0.001; stride length, *P* < 0.001; stride time, *P* < 0.001) groups.

**Figure 1:**
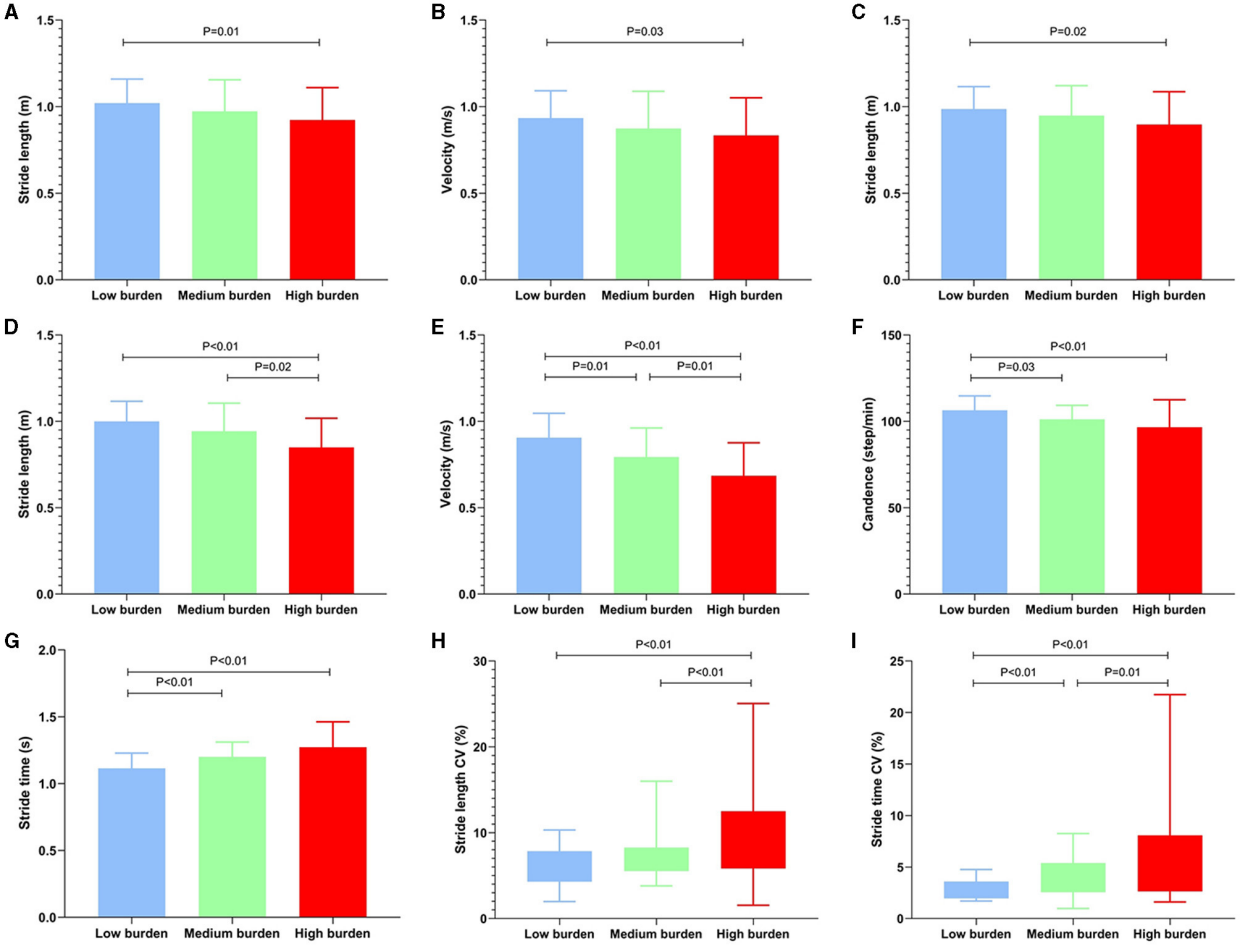
Significant differences among the total CSVD burden in basic parameters and its CV. The basic parameter values were represented by medians, while the CV was represented by the interquartile range. CSVD, cerebral small vessel disease; STW, single-task walking; DTW, dual-task walking; CV, coefficient of variation. **(A, B)** Differences of basic parameters among the groups during STW; **(C)** Differences of basic parameters among the groups during motor DTW; **(D–G)** Differences of basic parameters among the groups during cognitive DTW; **(H, I)** Differences of CV among the groups during cognitive DTW. The criterion for statistical significance was set at *P* < 0.05.

**Figure 2:**
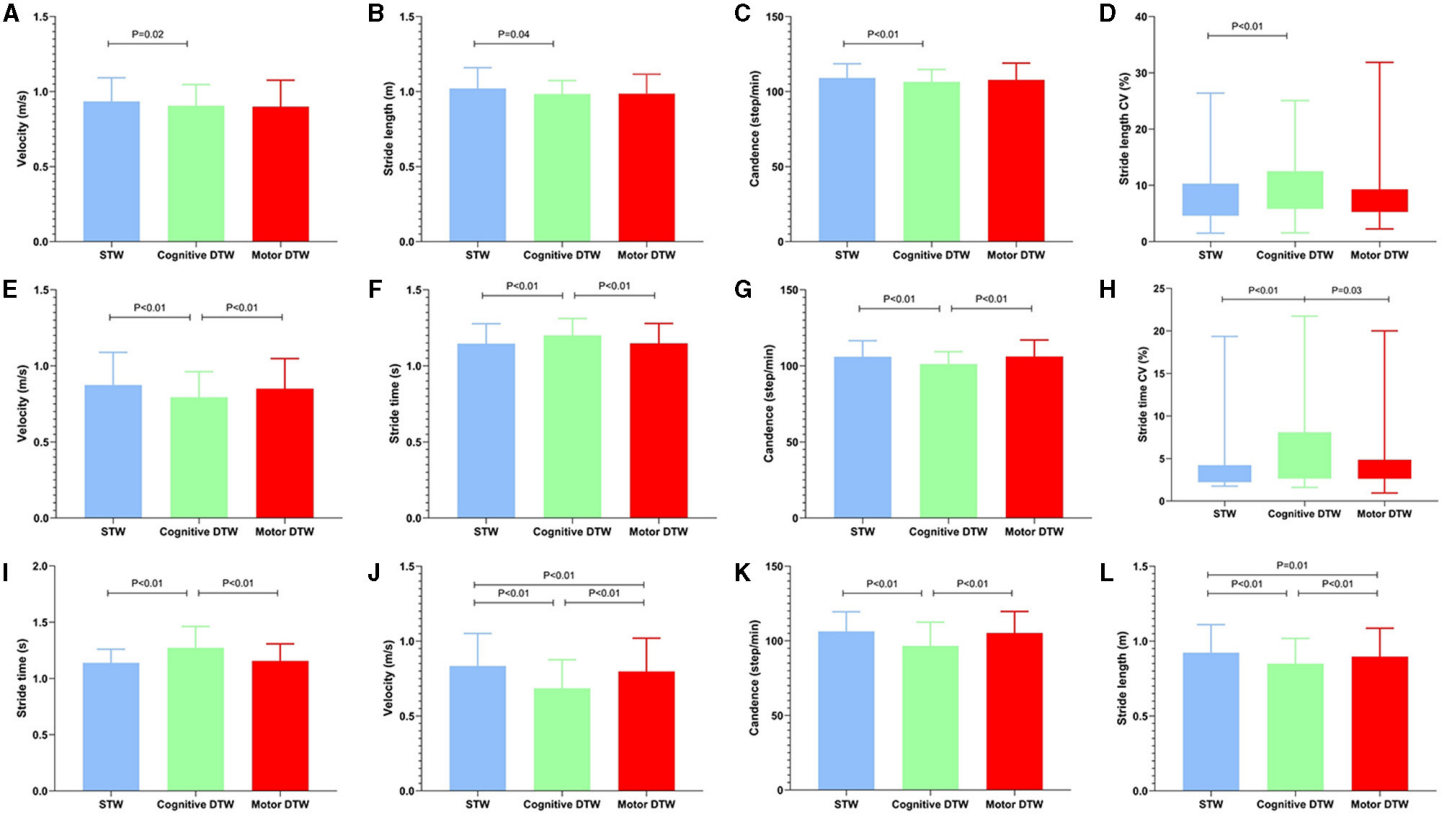
Significant intergroup differences in basic parameters and its CV during different walking patterns. The basic parameter values were represented by medians, while the CV was represented by the interquartile range. STW, single-task walking; DTW, dual-task walking; CV, coefficient of variation. **(A–C)** Discrepancy of basic parameters during different walking patterns in low burden group; **(D, H)** Discrepancy of CV during different walking patterns in high burden group; **(E–G)** Discrepancy of basic parameters during different walking patterns in medium burden group; **(I–L)** Discrepancy of basic parameters during different walking patterns in high burden group. The criterion for statistical significance was set at *P* < 0.05.

**Figure 3:**
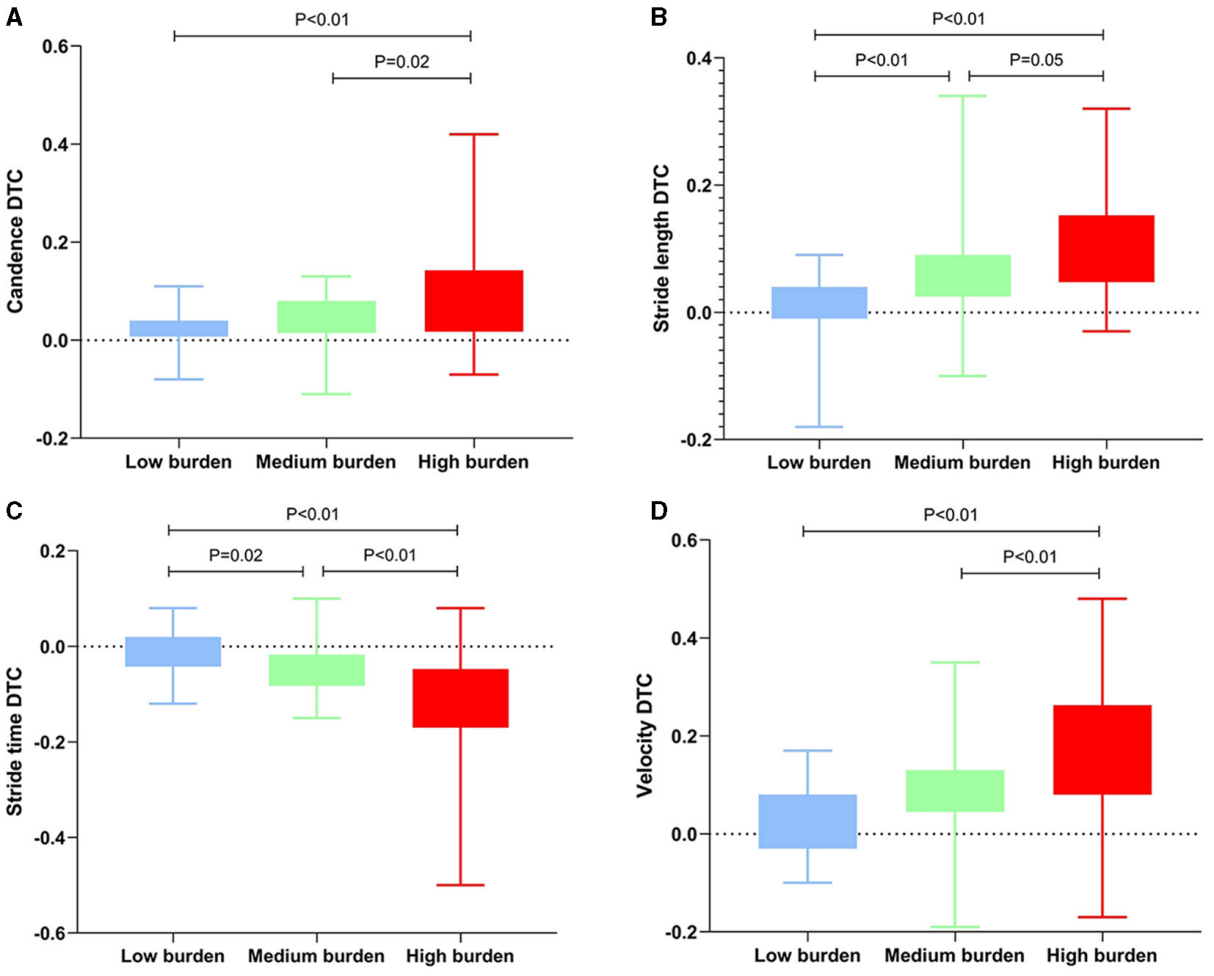
Significant differences among the total CSVD burden in DTC. The DTC was represented by the interquartile range. CSVD, cerebral small vessel disease; DTW, dual-task walking; DTC, dual task cost. **(A–D)** Differences of DTC among the groups during cognitive DTW. The criterion for statistical significance was set at *P* < 0.05.

**Figure 4:**
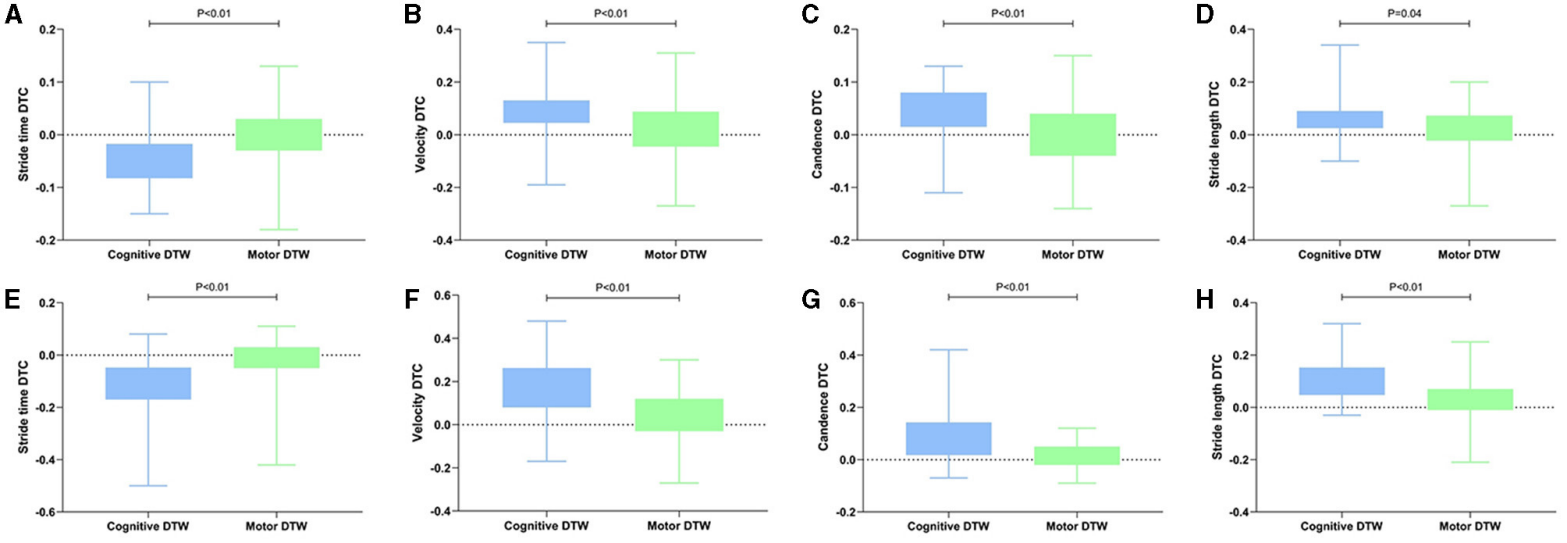
Significant intergroup differences in DTC during different walking patterns. The DTC was represented by the interquartile range. DTW, dual-task walking; DTC, dual task cost. **(A–D)** Discrepancy of DTC during different walking patterns in medium burden group; **(E–H)** Discrepancy of DTC during different walking patterns in high burden group. The criterion for statistical significance was set at *P* < 0.05.

The values of spatiotemporal gait parameters measured in both STW and DTW conditions showed in Tables 4, 5. The significant differences of *post-hoc* analysis showed in Figures 1–4.

**Table 4:** The value of spatiotemporal gait parameters in the condition of STW and DTW.

	**Group**		
	**Low burden (*****n*** = **30)**	**Medium burden (*****n*** = **34)**	**High burden (*****n*** = **62)**
**STW**			
Stride length (m)	1.021 ± 0.139	0.974 ± 0.182	0.923 ± 0.187^*^
Stride time (s)	1.096 ± 0.107	1.146 ± 0.131	1.138 ± 0.123
Candence (step/min)	109.103 ± 9.510	105.967 ± 10.580	106.315 ± 13.190
Velocity (m/s)	0.934 ± 0.158	0.874 ± 0.215	0.835 ± 0.217^*^
Stride length CV (%)	5.2 (4.3, 7.6)	6.6 (4.5, 9.5)	6.8 (4.6, 10.3)
Stride time CV (%)	2.5 (1.9, 3.8)	3.0 (1.8, 3.6)	2.8 (2.2, 4.3)
Candence CV (%)	3.9 (3.0, 5.4)	3.9 (3.1, 5.8)	4.1 (3.3, 6.8)
Velocity CV (%)	7.5 (5.7, 13.2)	8.1 (6.0, 12.3)	9.0 (7.0, 14.8)
**Cognitive DTW**			
Stride length (m)	0.982 ± 0.116^‡^	0.943 ± 0.162	0.849 ± 0.169^**^^†^^‡^^‡^
Stride time (s)	1.114 ± 0.115	1.200 ± 0.111^**^^‡^^‡^	1.272 ± 0.190^**^^‡^^‡^
Candence (step/min)	106.484 ± 8.283^‡^^‡^	101.184 ± 8.091^*^^‡^^‡^	96.575 ± 15.957^**^^‡^^‡^
Velocity (m/s)	0.905 ± 0.142^‡^	0.794 ± 0.168^**^^‡^^‡^	0.685 ± 0.19^**^^†^^†^^‡^^‡^
Stride length CV (%)	6.1 (4.3,7.8)	6.7 (5.5,8.3)	8.6 (5.8,12.5)^**^^†^^†^^‡^^‡^
Stride time CV (%)	2.8 (2.0,3.6)	3.7 (2.6,5.4)^**^^‡^^‡^	4.2 (2.7,8.1)^**^^†^^†^^‡^^‡^
Candence CV (%)	4.5 (3.3,5.7)	4.7 (3.6,6.1)	6.4 (4.0,9.2)
Velocity CV (%)	7.1 (6.1,9.5)	10.2 (6.3,12.9)	11.9 (8.7,18.0)
**Motor DTW**			
Stride length (m)	0.987 ± 0.129	0.949 ± 0.173	0.897 ± 0.189^*^^‡^
Stride time (s)	1.113 ± 0.157	1.148 ± 0.131	1.155 ± 0.152
Candence (step/min)	107.825 ± 11.135	106.102 ± 10.897	105.266 ± 14.466
Velocity (m/s)	0.899 ± 0.177	0.850 ± 0.198	0.798 ± 0.222^‡^^‡^
Stride length CV (%)	6.2 (4.7, 8.4)	6.9 (5.2, 9.1)	8.2 (5.3, 9.5)
Stride time CV (%)	2.8 (2.3, 4.1)	3.0 (2.4, 4.2)	3.0 (2.6, 4.8)
Candence CV (%)	4.0 (3.2, 4.7)	4.2 (3.2, 5.1)	4.6 (3.4, 7.9)
Velocity CV (%)	8.6 (6.8, 10.7)	9.6 (6.6, 11.6)	11.3 (7.7, 17.5)

STW, single-task walking; DTW, dual-task walking; CV, coefficient of variation.

Compare with Low burden ^*^P < 0.05, ^**^P < 0.01; Compare with Medium burden^†^P < 0.05, ^†^^†^P < 0.01; Compare with STW condition^‡^P < 0.05,^‡^^‡^P < 0.01.

**Table 5:** The value of dual task cost in the condition of STW and DTW.

	**Group**		
	**Low burden (*****n*** = **30)**	**Medium burden (*****n*** = **34)**	**High burden (*****n*** = **62)**
**Cognitive DTW**			
Stride length DTC	0.027 (−0.010, 0.039)	0.055 (0.023, 0.093)^**^^‡^	0.094 (0.045, 0.156)^**^^†^^‡^^‡^
Stride time DTC	−0.014 (−0.043, 0.017)	−0.055 (−0.086, −0.013)^*^^‡^^‡^	−0.083 (−0.168, −0.044)^**^^†^^†^^‡^^‡^
Candence DTC	0.024 (0.007, 0.042)	0.049 (0.013, 0.079)^‡^^‡^	0.067 (0.020, 0.144)^**^^†^^‡^^‡^
Velocity DTC	0.037 (−0.028, 0.078)	0.103 (0.015, 0.131)^‡^^‡^	0.150 (0.082, 0.266)^**^^†^^†^^‡^^‡^
**Motor DTW**			
Stride length DTC	0.018 (−0.021, 0.059)	0.024 (−0.025, 0.075)	0.028 (−0.010, 0.072)
Stride time DTC	−0.005 (−0.036, 0.025)	0.000 (−0.027, 0.027)	0.000 (−0.048, 0.025)
Candence DTC	0.003 (−0.025, 0.036)	−0.003 (−0.038, 0.039)	0.007 (−0.023, 0.047)
Velocity DTC	0.022 (−0.040, 0.061)	0.027 (−0.053, 0.080)	0.034 (−0.027, 0.118)

DTW, dual-task walking; DTC, dual task cost.

Compare with Low burden ^*^P < 0.05, ^**^P < 0.01; Compare with Medium burden^†^P < 0.05,^†^^†^P < 0.01; Compare with Motor DTW condition^‡^P < 0.05,^‡^^‡^P < 0.01.

### 3.3. Correlation between the severity of neuroimaging biomarkers and spatiotemporal gait measures

As shown in Table 6, there was a relationship between spatiotemporal gait measures and the CSVD burden. Regarding gait velocity, the total CSVD burden score showed a moderate negative relationship with speed and a positive correlation with its variability or DTC in the cognitive DTW condition; however, it was not correlated with any of the parameters in the STW or motor DTW condition. Similar results were observed for WMHs; however, they had a relatively smaller correlation coefficient. Lacune infarctions were moderately correlated with cognitive DTW velocity and its DTC; moreover, the correlation coefficient was similar to that for WMHs. However, there was no relation with the variability. Additionally, microbleeds were not correlated with any of the spatiotemporal measures.

**Table 6:** Relationship between gait and neuroimaging burden.

	**STW**	**Cognitive DTW**	**Motor DTW**	**DTC of cognitive DTW**	**DTC of motor DTW**	**STW variability**	**cognitive DTW variability**	**Motor DTW variability**
**Velocity R (** ***P*** **)**								
Total score	−0.192 (0.031)	−0.474^**^ (< 0.001)	−0.201 (0.024)	0.493^**^ (< 0.001)	0.067 (0.459)	0.172 (0.055)	0.381^**^ (< 0.001)	0.223 (0.012)
WMH	−0.167 (0.062)	−0.367^**^ (< 0.001)	−0.236 (0.008)	0.348^**^ (< 0.001)	0.181 (0.042)	0.166 (0.063)	0.337^**^ (< 0.001)	0.260 (0.003)
LI	−0.136 (0.130)	−0.330^**^ (< 0.001)	−0.148 (0.097)	0.316^**^ (< 0.001)	0.098 (0.274)	−0.109 (0.226)	0.191 (0.032)	−0.019 (0.083)
CMB	−0.031 (0.727)	−0.227 (0.011)	−0.066 (0.466)	0.305 (0.001)	0.068 (0.435)	0.015 (0.865)	0.116 (0.197)	0.083 (0.345)
**Cadence R (** ***P*** **)**								
Total score	−0.082 (0.359)	−0.331^**^ (< 0.001)	−0.099 (0.268)	0.335^**^ (< 0.001)	0.037 (0.677)	0.133 (0.139)	0.301^**^ (< 0.001)	0.186 (0.063)
WMH	−0.088 (0.330)	−0.344^**^ (< 0.001)	−0.144 (0.107)	0.366^**^ (< 0.001)	0.161 (0.071)	0.143 (0.109)	0.234 (0.008)	0.212 (0.017)
LI	−0.088 (0.325)	−0.211 (0.018)	−0.087 (0.333)	0.139 (0.121)	−0.021 (0.896)	0.069 (0.440)	0.103 (0.252)	−0.009 (0.920)
CMB	−0.053 (0.557)	−0.097 (0.280)	−0.010 (0.913)	0.166 (0.063)	0.076 (0.398)	−0.083 (0.357)	0.050 (0.575)	−0.029 (0.747)
**Stride length R (** ***P*** **)**								
Total score	−0.176 (0.048)	−0.404^**^ (< 0.001)	−0.203 (0.036)	0.506^**^ (< 0.001)	0.085 (0.346)	0.174 (0.049)	0.365^**^ (< 0.001)	0.147 (0.101)
WMH	−0.124 (0.166)	−0.224 (0.012)	−0.178 (0.041)	0.298 (0.001)	0.056 (0.53)	0.120 (0.179)	0.256 (0.004)	0.157 (0.079)
LI	−0.133 (0.139)	−0.345^**^ (< 0.001)	−0.153 (0.088)	0.296 (0.001)	0.038 (0.672)	0.072 (0.425)	0.193 (0.030)	−0.025 (0.782)
CMB	−0.073 (0.419)	−0.186 (0.037)	−0.080 (0.371)	0.324^**^ (< 0.001)	0.112 (0.211)	0.054 (0.547)	0.278 (0.002)	0.043 (0.634)
**Stride time R (** ***P*** **)**								
Total score	0.115 (0.199)	0.383^**^ (< 0.001)	0.110 (0.218)	−0.475^**^ (< 0.001)	−0.022 (0.448)	0.135 (0.132)	0.302^**^ (< 0.001)	0.124 (0.166)
WMH	0.079 (0.378)	0.315^**^ (< 0.001)	0.155 (0.083)	−0.413^**^ (< 0.001)	0.162 (0.070)	0.143 (0.111)	0.273 (0.002)	0.123 (0.170)
LI	0.119 (0.186)	0.233 (0.009)	0.120 (0.182)	−0.200 (0.025)	−0.024 (0.787)	0.117 (0.192)	0.159 (0.076)	0.197 (0.027)
CMB	−0.029 (0.748)	0.087 (0.330)	0.001 (0.992)	−0.214 (0.016)	−0.096 (0.287)	0.076 (0.397)	0.050 (0.576)	−0.092 (0.306)

CMB, cerebral microbleeds; DTC, dual-task cost; DTW, dual-task walking; LI, lacunar infarction; STW, single-task walking; WMH, white matter hyperintensity.

R value: 0.1–0.3, small; 0.3–0.5, medium; 0.5, large. After Bonferroni correction, the criterion for statistical significance was set at P < 0.0004^**^.

Regarding cadence, the total CSVD burden score and WMHs showed a moderate negative relationship with the cadence in the cognitive DTW condition and a positive relationship with its DTC. The correlation coefficients were similar. Contrastingly, only the total CSVD burden score was significantly related to the variability of cadence. Microbleeds and lacune infarctions were not correlated with cadence parameters.

Regarding stride length, the total CSVD burden had a moderate correlation with stride length and its DTC or variability in the cognitive DTW condition but not in the STW and motor DTW conditions. However, WMHs were not correlated with stride length and its DTC or variability. Contrastingly, lacune infarctions showed a moderate negative correlation with stride length, whilst microbleeds showed a positive correlation with the DTC of stride length in the cognitive DTW condition.

Finally, the total CSVD burden had a moderate correlation with stride time and its DTC or variability in the cognitive DTW condition. Similar to that in speed, WMHs were correlated with stride time and its DTC, but not its variability, in the cognitive DTW condition. WMHs had a smaller correlation coefficient than that of the total CSVD burden score. Similarly, lacune infarctions and microbleeds were not correlated with any of the parameters for stride time.

## 4. Discussion

We observed among-group differences in all gait parameters during cognitive DTW. However, for STW and motor DTW, only some parameters showed differences between the low-burden and high-burden groups. In addition, the total CSVD burden had the strongest relationship with gait parameters in the cognitive DTW condition compared with any single neuroimaging index.

### 4.1. Gait characteristics during STW

Previous studies on older patients with Alzheimer's disease have discussed the correlation between WMH and gait characteristics during STW. They indicated that only among-group stride length differences were detected according to the degree of WMH (37). Another study revealed that CSVD-related white matter alterations were significantly correlated with reduced stride length in individuals with pure CSVD (8). Similarly, another study showed that spatial, but not temporal, gait features were correlated with the WMH degree in the STW condition (38). A Chinese cross-sectional study on patients with CSVD showed that Fazekas scores were associated with gait parameters, including the step speed and length, but not frequency, in the STW condition (39). These findings could be attributed to the fact that CSVD commonly damages the integrity of subcortical connexions (40). Spatial gait parameters are primarily controlled by cortical and subcortical areas, specifically neurons connecting in the frontal cortex and basal ganglia. Contrastingly, temporal features might be more dependent on mechanisms in the brainstem and spinal cord (41, 42).

Our findings are inconsistent with those of the previous reports. Although speed and stride length differed between the low-burden and high-burden groups, the total CSVD burden or WMH was not significantly correlated with spatial gait parameters following the Bonferroni correction. This indicates that gait parameters obtained in the STW condition are not sensitive indicators for the severity of CSVD.

### 4.2. Spatial and temporal gait features during DTW

DTW is a complex task requiring normal cognitive functions; accordingly, performance deterioration in DTW is indicative of competition for attentional resources (43). CSVD severity is negatively correlated with the availability of attentional resources (44). To this end, gait performance deteriorates when more demanding tasks are simultaneously implemented. However, the specific associations between CSVD biomarkers and gait performance in the DTW condition remain unclear.

#### 4.2.1. Characteristics of gait during motor DTW

A previous study (38) showed that WMH volume had a negative association with velocity and stride length in the motor DTW condition, which is inconsistent with our findings. In our study, although stride length differed between the low-burden and high-burden groups in the motor DTW condition, total CSVD burden and WMH were not correlated with spatial gait parameters after Bonferroni correction.

These findings are consistent with the task prioritisation model (45), which suggests that individuals prioritise complex tasks in case of competition for attentional resources. Walking requires mainly somatosensory central processing and somatosensory responses; moreover, secondary motor tasks also require additional manual responses (46). Therefore, both tasks have similar complexities, and the secondary motor task does not take up extensive neural resources. Accordingly, gait parameters in the motor DTW condition are not affected in the early disease stage.

Additionally, the correlation coefficients of the total CSVD burden score or WMH were similar between the motor DTW and STW conditions. Taken together, gait parameters in the motor DTW condition cannot effectively distinguish the severity stratification of disease among patients with CSVD.

#### 4.2.2. Gait characteristics during cognitive DTW

Hairu et al. (38) discovered that temporal and parietal WMH volumes were negatively correlated with stride length during cognitive DTW. Another study indicated that deep WMHs were significantly correlated with gait speed in the DTW condition (5, 47), and executive function mediated this relationship. Patients with pure CSVD have shown deficits in all spatiotemporal gait parameters in the semantic DTW condition (8). Additionally, compared with healthy individual, CSVD patients showed significant deteriorate in stride time CV during cognitive DTW (9, 48). In our study, almost all gait parameters showed differences among groups that were stratified according to total CSVD burden score. These findings were consistent with those in the correlation analysis. The total CSVD burden score was correlated with temporal and spatial parameters and their variabilities in the DTW condition. Moreover, the DTCs of basic gait parameters showed an identical performance with basic parameters. This can be attributed to the interlinking between brain networks responsible for pace control and those responsible for cognitive task performance (9), leading to competition for the same resources and, thus, impairment of all gait characteristics (8).

Pantoni reported that among several neuroimaging biomarkers, WMHs in specific brain regions had the strongest correlation with gait disturbance (12). Contrastingly, we found that the total CSVD burden but not WMHs had the strongest correlation with gait disturbance. Specifically, the variability of spatiotemporal parameters was associated with total CSVD burden but not WMHs; moreover, the correlation coefficient of WMHs was lower than that of the total CSVD burden score for all gait parameters except cadence. This indicates that the total CSVD burden score is a stronger predictor of gait performance than any single biomarker. Notably, only a few gait parameters were associated with microbleeds and lacunar infarcts, indicating the poor predictive utility of these biomarkers for gait abnormalities.

### 4.3. Performance differences between cognitive and motor DTW

Hairu et al. (38) reported that in patients with Alzheimer's disease, motor DTW resulted in greater deterioration of gait characteristics (velocity, cadence, and stride length) than cognitive DTW. Similarly, Cherng et al. (46, 49) found that in children with developmental coordination disorder, motor DTW resulted in greater interference of gait parameters than cognitive DTW, as indicated by decreased velocity, cadence, stride length, and double limb support. Contrastingly, we found that gait parameters in the cognitive, but not motor, DTW condition were significantly correlated with the disease severity.

According to Wickens, attentional resources are not unitary but are divided into various pools, which are “defined by a three-dimensional metric consisting of the stages of processing, codes of perceptual and central processing, and modalities of input and response” (50). Walking requires mainly somatosensory central processing and somatosensory responses. In addition, the secondary motor task also requires additional manual responses. Contrastingly, a cognitive secondary task will require further executive functional and verbal central processing as well as vocal responses (46), with limited opportunities for compensatory mechanisms. Therefore, in cognitive DTW, more brain regions may be active, and the remaining resources do not meet the demands of gait control. Accordingly, a cognitive task greatly interferes with walking, leading to obvious gait abnormalities in the early disease stage.

In our study, the DTC in the cognitive DTW condition was significantly higher than that in the motor DTW condition, suggesting that parameters in the cognitive DTW condition could better predict abnormal gait in individuals with CSVD. This finding is inconsistent with previous literature (38, 46, 49), which could be attributed to differences in the examined diseases. Specifically, Alzheimer's disease mainly causes grey matter damage, whilst CSVD mainly affects cortical and subcortical connecting fibres (38). Furthermore, developmental coordination disorder is a movement disorder; accordingly, the motor DTW condition yields more obvious differences (46).

Taken together, patients with CSVD showed significant gait deterioration, especially in the cognitive DTW condition. Our conclusion has been substantiated by an increasing body of evidence. A literature review spanning from 1997 to 2017 revealed that gait changes associated with cognitive DTW may serve as a potential marker for nervous system diseases (51). Therefore, in daily life, patients should reduce the load of secondary tasks while walking, especially cognitive tasks. However, the underlying brain network mechanisms during DTW remain unclear. Future studies should use diffusion tensor imaging to observe changes in white matter fibre during DTW.

Furthermore, despite the prevalence of gait disorders and falls among patients with CSVD (11, 12, 15), a definitive cure remains elusive. Previous studies have indicated that cognitive dual-task training is both safe and effective in improving various spatiotemporal gait parameters (52). Additionally, another systematic review confirmed that cognitive DTW training improves balance and reduces the incidence of falls in older adults (53). Against this backdrop, we have substantiated the significance of cognitive DTW for patients with CSVD. We speculate that CSVD patients would also derive benefits from dual-task training, including improved exercise performance and reduced fall incidence. However, as no studies have investigated the impact of dual-task training in CSVD patients, a prospective cohort study is necessary to explore the implications of dual-task training in the future.

### 4.4. Study limitations

This study has some limitations. First, the sample size was relatively small; accordingly, a large-scale multicentre longitudinal study is warranted to verify our findings. Second, the patients with CSVD had different clinical features. Therefore, there was heterogeneity in the participants of our study, which might have caused bias in the results. Third, the neuroimaging assessment was not extensively comprehensive and accurate. Specifically, neuroimaging findings were semi-quantitatively evaluated, which could have introduced systematic errors. The use of quantitative technology could better demonstrate among-group differences and correlations, especially during cognitive DTW. Fourth, although the sample entropy, asymmetry and phase coordination index might be good indicators, we did not include it in our study. In the future, our intention is to increase the sample size to further investigate the role of these parameters in the early diagnosis and management of CSVD.

## 5. Conclusions

Parameters in the cognitive DTW condition may be more appropriate than those in the motor DTW condition for the evaluation of gait abnormalities in patients with CSVD. Moreover, the total CSVD burden score might have a better predictive utility than any single neuroimaging marker. Patients with CSVD, especially those with moderate-to-severe disease, should reduce the load of secondary tasks while walking in daily life, especially cognitive tasks.

## Data availability statement

The raw data supporting the conclusions of this article will be made available by the authors, without undue reservation.

## Ethics statement

The studies involving humans were approved by Academic Ethics Committee of the Biological Sciences Division of the Seventh Medical Centre of the People's Liberation Army General Hospital (Beijing, China). The studies were conducted in accordance with the local legislation and institutional requirements. The participants provided their written informed consent to participate in this study.

## Author contributions

CX: Writing—original draft. HX: Writing—original draft. TL: Writing—review & editing. YD: Writing—review & editing. HZ: Writing—review & editing. YH: Writing—review & editing.
